# Pathological margins and advanced cutaneous squamous cell carcinoma of the head and neck

**DOI:** 10.1186/s40463-019-0374-3

**Published:** 2019-10-25

**Authors:** T. J. Phillips, B. N. Harris, M. G. Moore, D. G. Farwell, A. F. Bewley

**Affiliations:** 1 Department of Otolaryngology-Head and Neck Surgery, UC Davis, Sacramento, California USA; 2 Division of Head and Neck Surgery, Kingston Health Science Center, Queen’s University, 2nd Floor Murray Building, Hotel Dieu Hospital, Bagot Street, Kingston, Ontario Canada

## Abstract

**Objective:**

The recommended treatment for cutaneous squamous cell cancer (CuSCC) of the head and neck is Mohs surgical excision or wide local excision. Excision is recommended to a gross surgical margin of 4–6 mm however this is based on limited evidence and specify a goal histologic margin. The objective of this study was therefore to examine the reported histological margin distance following WLE of advanced CuSCC and its association with recurrence and survival.

**Study design:**

Retrospective database review.

**Setting:**

All patients included received treatment at UC Davis Department of Otolaryngology-Head and Neck Surgery and/or Radiation Oncology in Sacramento, California.

**Subjects and methods:**

The patients included were treated for advanced CuSCC with primary surgery with or without adjuvant therapy. Kaplan Meier survival curves with log rank analysis were then performed to compare 5-year recurrence free survival, and disease-specific survival for patients with different margin distances.

**Results:**

Total number of subjects was 92. The overall 5-year DSS and RFS was 68.8 and 51.0% respectively. When the pathological margin distance was ≥5 mm, 5-year disease specific survival was improved when compared to margin distance less than 5 mm (94.7 vs 60.7 *p* = 0.034).

**Conclusion:**

The findings of this study suggest that a histologic margin of at least 5 mm may increase survival in advanced head and neck CuSCC patients.

## Introduction

Cutaneous squamous cell cancer (CuSCC) is the second most common cancer, behind basal cell carcinoma with an incidence of roughly 2 million cases per year in the United States [1]. The majority of these cancers are small and have an excellent cure and survival rate of 90–99% [1]. However, CuSCC if neglected or aggressive does have the propensity to grow and destroy local structures along with the ability to send local and distant metastasis. CuSCC also has the tendency for more aggressive behavior in patients who are immunocompromised [2]. Management of CuSCC depends on several factors including the size, location, metastatic disease, and patient factors. In general, excision of the CuSCC with a normal tissue margin is the standard treatment [3].

The gold standard of care for CuSCC and other skin cancers is Moh’s surgical excision, which has a reported cure rate of 96–99% [3, 4]. However, Moh’s surgical resection is not always possible. A second, highly effective method of resection is wide local excision (WLE). This too has a high cure rate of 90–97% and can often be used when Mohs surgery is not indicated or available [3].

The treatment of CuSCC is outlined in the NCCN guidelines. For “low-risk” CuSCCs of the head and neck the recommended gross margins on surgical resections are 4–6 mm [5]. Obtaining this margin in the head and neck can be challenging given the proximity of vital anatomy. For patients with high-risk features the guidelines recommend taking a larger margin, however the exact increase is not defined. The margin of 4–6 mm is based on a single prospective non-randomized study that used Moh’s technique. It recommended 4 mm for tumors less than 2 cm in greatest dimension and 6 mm for those greater than 2 cm. It did not examine whether a 4–6 mm margin via WLE was equivalent to a 4-6 mm margin in Mohs nor did it study specific body site location [4]. This study also describes only gross margins and not histologic margins.

Histologic margin goals for CuSCC are not discussed within the NCCN for CuSCC and there are no previous papers that have addressed this topic. For oral mucosal SCC the importance of histological margin distance is well established. Loree et al orginally determined that a histological margin of 5 mm or more results in greater survival and less local recurrence than close or positive margins [6, 7]. It is also recommended that taking a gross 1 cm margin of mucosa will typically yield the recommended 5 mm histologic margin [8]. The precise relationship between gross margin and histologic margin is not known for CuSCC.

The objective of this study was to examine the reported histologic margin distance following WLE of advanced CuSCC and its association with recurrence and survival.

## Methods

This study was designed as a retrospective chart review. Ethics approval was obtained from the University of California ethics board. An established CuSCC database developed at UC Davis Otolaryngology-Head & Neck Surgery was used to identify patients [9]. The database contained patients treated from 1998 to 2014 for CuSCC of the head and neck. All patients undergoing surgical treatment with or without adjuvant therapy for curative intent were included in the study. The database contained patient information (age, sex, and immunologic status) and data regarding tumor characteristics (primary site, DOI, diameter, lymphovascular invasion, PNI, presence of regional nodal disease, histologic differentiation, adjuvant therapy, margin status, and whether tumors were recurrent on presentation). Patients were considered immunocompromised if they were HIV+, on immunosuppression drugs for transplantation, or undergoing chemotherapy. The numbers of each type of immunosuppressed patient were not recorded. All patients were treated in a head and neck oncology practice and consequently all had advanced stage (III & IV) disease as defined by primary tumor size ≥ 4 cm, deep invasion (beyond subQ fat or > 6 mm), bone erosion, PNI, or presence of nodal disease.

For the surgical technique, a wide margin was marked around the tumor site. The extent of the tumor was based on palpation and visual inspection by the surgeon. The gross margin taken by the surgeon was not recorded in the database, therefore only the histologic margin recorded by the pathologist was used in this study. Intraoperative frozen sections were taken to confirm clear margins, as per NCCN guidelines. The frozen sections were taken “off the patient” rather than off the specimen. The exact method for analyzing the margins by the pathologist is unknown, due to the retrospective nature of this study.

Statistical analyses were performed with SPSS 23.0 (SPSS Inc., Chicago, Illinois). A chi square analysis was conducted to assess for a correlation between outcome and margin status. The following factors were examined: recurrent cancer, LVI, PNI, immunosuppression, poorly differentiated SCC. Kaplan Meier survival curves with log rank analysis were performed to assess 5-year recurrence free survival (RFS) and disease free survival (DSS) at different margin distances.

## Results

**Fig. 1:**
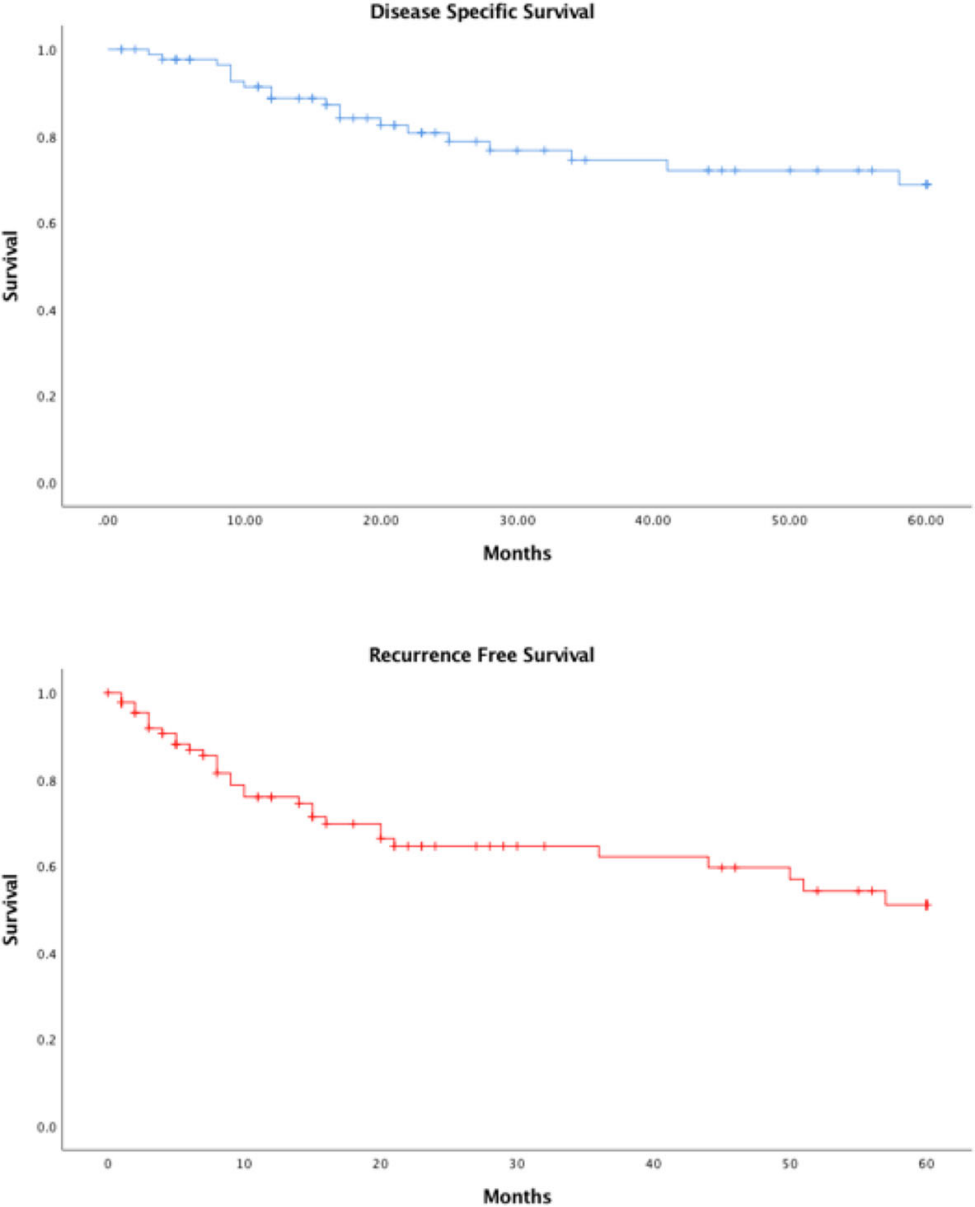
The 5-year disease specific survival (68.8%) and recurrence free survival (51.0%) for all patients (*N* = 92)

A total of 232 patients were entered in the database. 92 (40%) of these had a recorded histologic margin distance. The remaining patients either had clear margins without distance recorded (45%) or no primary cancer remaining (15%). Demographic and tumor characteristics are summarized in Table 1. Of the patients included in the study the average age was 69 and the majority of participants were male (85%) (Table 1). The overall 5-year disease specific survival and recurrence free survival was 68.8 and 51.0% respectively (Fig. 1).

**Table 1:**
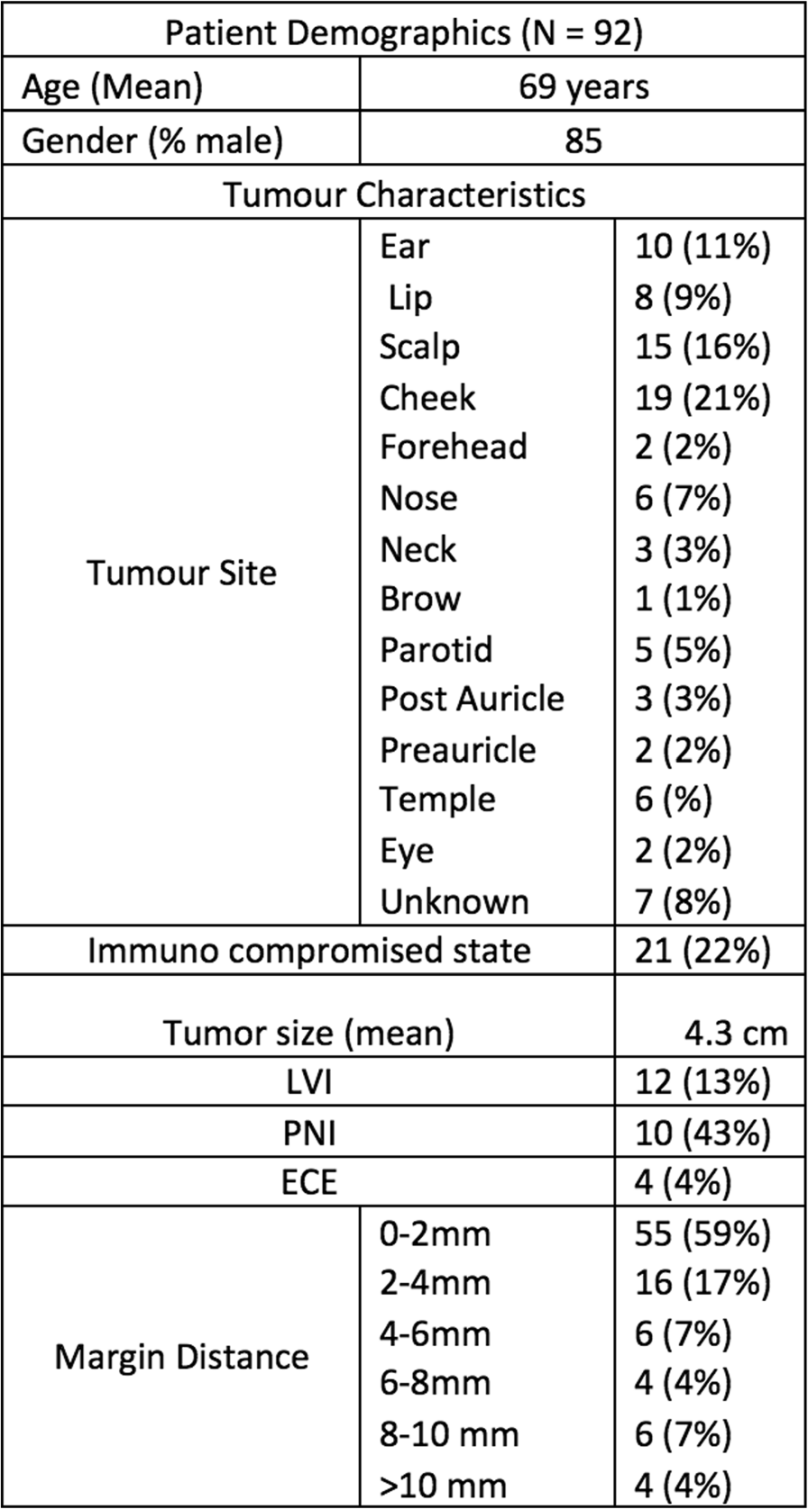
Patient Demographics and Tumor Characteristics

**Fig. 2:**
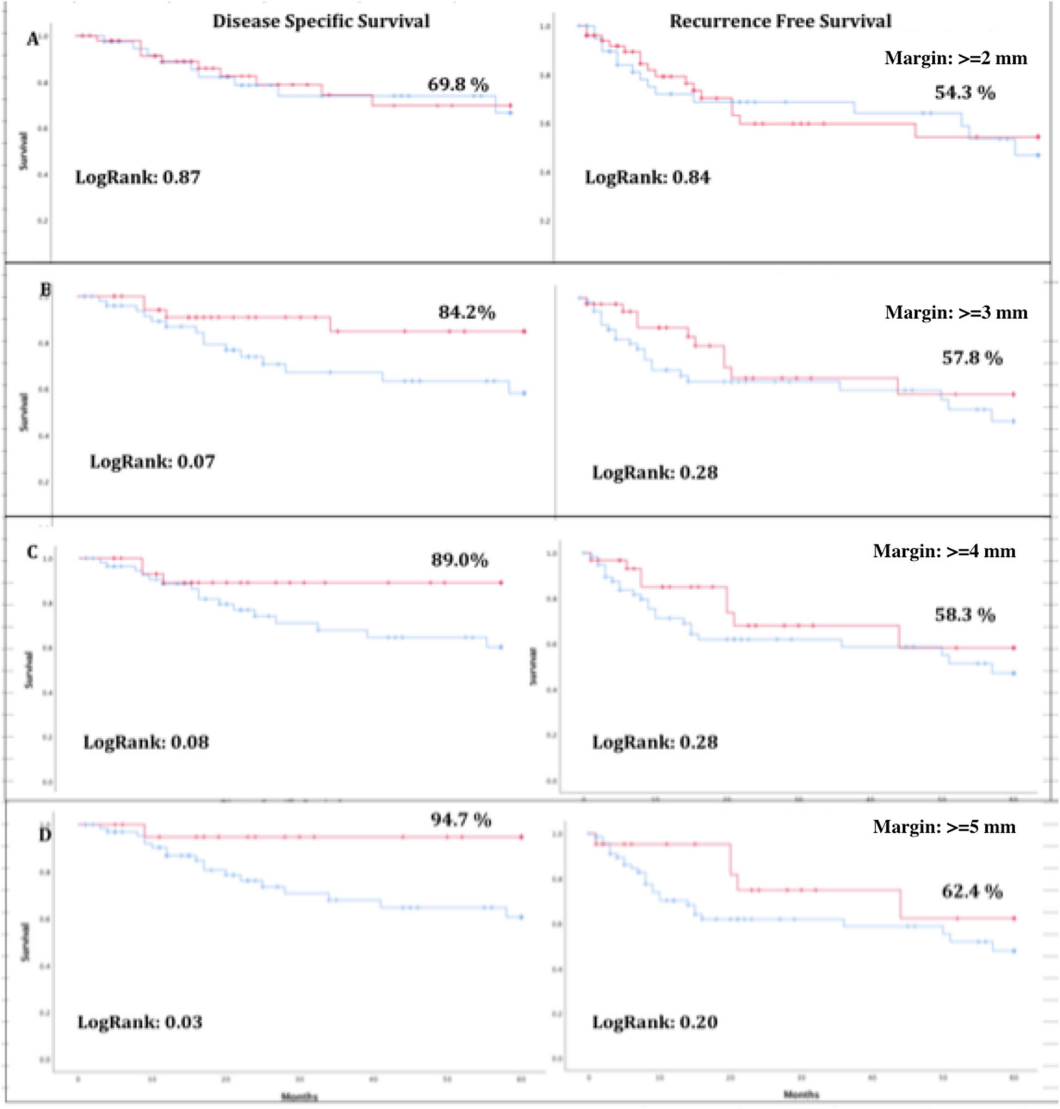
Change in DSS and RFS as margin distance increases

For patients with recorded margins the average margin distance was 3.6 mm. There was 55 patients with 0–2 mm margins (59%), 16 with 2-4 mm (17%), 6 with 4-6 mm (7%), 4 with 6-8 mm (4%), 6 with 8–10 mm margin (7%), and 4 with > 10 mm margins (4%). Patients were then divided into groups based on the distance of their closest margin (< 1 vs ≥ 1 mm, < 2 vs ≥ 2 mm, … .. < 5 vs ≥ 5 mm). Kaplan Meier survival analysis was then used to compare 5 year RFS and 5 year DSS between these groups for each margin threshold (Fig. 2). At a margin distance of ≥5 mm we observed significantly improved DSS (94.7 vs 60.7 *p* = 0.034) and a non-significant trend towards improved RFS (62.4 vs 47.9% *p* = 0.20).

A Chi-square analysis did not show any significant correlation between margin distance and recurrent cancer, LVI, PNI, immunosuppression, and poorly differentiated CuSCC.

## Discussion

There is a paucity of evidence regarding margin goals for resection of CuSCC. The findings of this study demonstrate that histologic margins of 5 mm or more may increase survival in patients undergoing WLE for advanced CuSCC of the head and neck. Examining histologic margins in CuSCC in relation to survival has not been examined before, however the results reflect the recommendations for oral mucosa SCC [6, 7].

As previously mentioned the NCCN guidelines recommend a gross margin of 4–6 mm for CuSCC [5]. This was based on a single study by Brodland and Zitelli from 1992 that used Mohs technique [4]. With Mohs technique the surgeon performs both gross and histologic assessment of the tumor at the time of resection. With WLE, surgeons rely on visual and tactile feedback in taking appropriate margins with selected use of frozen section. There have been no studies examining whether a 4–6 mm margin via WLE is equivalent to a 4–6 mm margin using Mohs. The relationship of the gross margin compared to the final histologic margin after WLE for CuSCC has not been studied either. One study did specifically examine margin shrinkage after Moh’s surgical excision and found a decrease in distance by 10% for head and neck specimens [10]. Also, it is known that there is roughly a 10–17% shrinkage of skin specimens after resection. Whether this rate of shrinkage can be applied to margin distance after WLE has not been assessed.

This study used closest histologic margins, meaning that the distance reported by the pathologist after the specimen underwent pathological processing. Histologic margin goals are not addressed by the NCCN for CuSCC and this is not a topic that has been previous studied. For mucosal disease an established histologic margin of 5 mm is the recommendation to ensure increased disease survival and decreased locoregional recurrence [7]. The shrinkage rate of oral mucosal specimens have also been established, and having a 1 cm margin intraorally should allow for a 5 mm histologic margin [8]. Further prospective studies need to be performed to establish this relationship for CuSCC.

A strength of this study is that it assessed CuSCC only of the head and neck. Establishing treatment recommendations specific to the head and neck is important as a high proportion of skin cancers develop on the head and neck, and the anatomy and lymphatic drainage of the head and neck is unique. Also, given the functional and aesthetic importance of the head and neck, appropriate margin distance is paramount to minimizing the morbidity of resection.

This study also distinguishes itself as it examined advanced head and neck CuSCC. All the patients in the present study were considered advanced either due to size, location, recurrence, or locoregional metastatic spread. This study suggests that even with advanced disease, a > 5 mm histologic margin results in greater survival than a < 5 mm margin.

Another limitation within the study is that only 40% of patients within the database had reported margins. This is possibly due to reporting standards changing for pathology. Therefore, it is possible that the 40% included in the study could not be a representative sample.

One final point that was difficult to explain was why DSS was associated with margin distance but RFS was not. One would assume that patients who recur are more likely to die of their disease and as such they should be tightly associated. The correlation between the margin status and DSS may be stronger than with RFS because there was other prognostic factors that had a significant influence on RFS, which “dilutes” this correlation.

There are multiple directions that future studies could take. A comparison of gross surgical margins, final histologic margins and oncologic outcomes would certainly be informative. This would help to determine what gross margins are required to obtain a pathologic margin goal of 5 mm or more. There is hope that data from outside centers will eventually be added to the study to provide more robust data.

Further work could also include examining high risk patients. These include immunocompromised patients such as transplant patients. This group of patients have a much higher risk of developing CuSCC, have more aggressive cancers, and worse outcomes. The NCCN guidelines suggest taking a “larger margin” for these patients, but do not specify how much larger [5]. The European guidelines suggests 10 mm, but this is based mainly on expert opinion [11]. It would be beneficial to try to determine an appropriate margin distance in this at-risk group of patients in order to guide management.

In conclusion, this study suggests that a 5 mm or greater histologic margin may increase survival in head and neck CuSCC patients, which correlates with the recommendations for Oral SCC. Further prospective studies are required to provide appropriate guidelines for managing advanced or high risk CuSCC.

## Data Availability

The datasets during and/or analyzed during the current study available from the corresponding author on reasonable request.
